# Gonorrhœa and Homœopathy

**Published:** 1891-02

**Authors:** J. C. White

**Affiliations:** Portchester, N. Y.


### GONORRHŒA AND HOMŒOPATHY.

Editors of The Homœopathic Physician:—While the gonorrhœa subject is being ventilated through the medium of your journal I shall look anxiously for light on the subject of treatment of the poison—the disease *per se*. I confess to a feeling of disappointment in reading Dr. Dever's article in the November number. Every careful student of Homœopathy is supposed to be able to treat a chronic case—where the discharge is kept up by some constitutional dyscrasia—such symptoms as then guide us in their treatment, are not in the "foreground," and are seldom attainable in the acute stage. How are we to treat or to cure a typical case of acute gonorrhœa? My own experience is negative. I have never seen a recovery under homœopathic treatment in less than six or eight weeks' time. I am satisfied that unaided nature does as well where the subject is in good health. In a typical case of acute gonorrhœa of an otherwise healthy subject are we not absolutely reduced to clinical indications? If so, what remedies kill or neutralize the poison?

Are not injections of an homœopathic solution of Mercury equivalent to the dose by the mouth, and has it any preference to the Mercury given by the mouth? I have as yet seen no proof that Mercury so used can cause a suppression of gonorrhœa, in the sense in which Dr. Skinner uses the word suppression. The case he sites was suppressed by a powerful astringent —equivalent to Tannic or Gallic acid—ignorantly and evidently without expectation of curing or of neutralizing the virus. I have never been cognizant of such treatment either in the new or the old school. Give us light, and if we may choose, we prefer it without ridicule. God's light shines beneficently. Let those who first learn the truth reflect it accordingly.

J. C. White.

Portchester, N. Y., December 24th, 1890.

